# Supplementary material for: A cortico-collicular pathway for motor planning in a memory-dependent perceptual decision task
Source: Nat Commun. 2021 May 11;12:2727. doi: 10.1038/s41467-021-22547-9 (PMC8113349; doi:10.1038/s41467-021-22547-9)
Supplement: Supplementary file 1 — Supplementary Information [file 41467_2021_22547_MOESM1_ESM.pdf]

Supplementary Information

A cortico-collicular pathway for motor planning in a memory-dependent perceptual decision task

Chunyu A. Duan[1,†,*], Yuxin Pan[1,2,†], Guofen Ma[3], Taotao Zhou[1], Siyu Zhang[3], Ning-long Xu[1,2,4,*]

*Correspondence to: Chunyu A. Duan, cduan@ion.ac.cn and Ning-long Xu, xunl@ion.ac.cn.

†These authors contributed equally to this work.

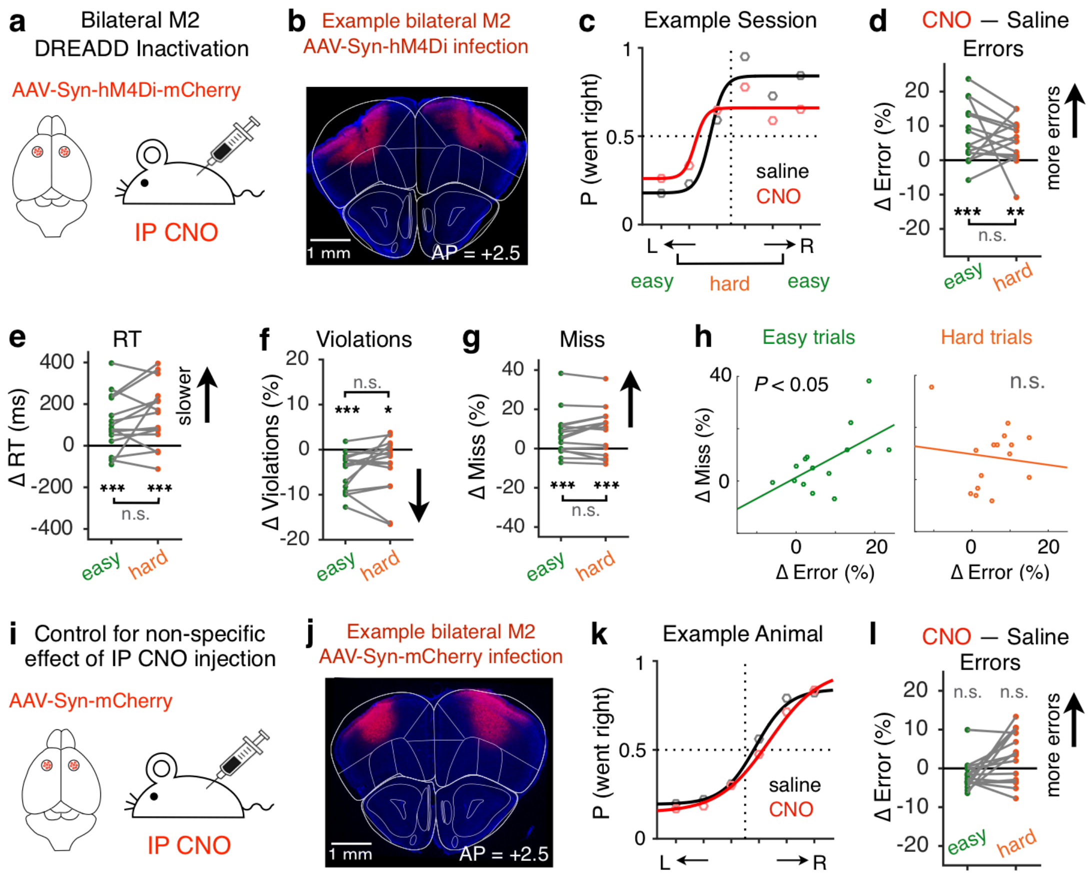

Supplementary Fig. 1 | Chemogenetic inactivation of M2 during behavior. a Design for bilateral M2 DREADD inactivation. **b** Example histology of bilateral M2 infection for chemogenetic inactivation. **c** Example M2 inactivation session (red) compared to saline control session (black) for one animal. Dots, data; lines, sigmoid fit. **d** Difference in error rate between M2 inactivation sessions (n=15) and saline control sessions (n=15) for easy (green) and hard (orange) trials. Gray lines connect data from the same session. ***$P = 4\times10^{-4}$ for easy trials; **$P = 0.0016$ for hard trials; n.s. $P = 0.35$, two-sided bootstrap or permutation tests. **e** Difference in RT, similar to **d**. ***$P = 8\times10^{-4}$ for easy trials; ***$P = 4\times10^{-4}$ for hard trials; $P = 0.11$ for easy versus hard trials, two-sided bootstrap or permutation tests. **f** Difference in violation rate, similar to **d**. ***$P = 4\times10^{-4}$ for easy trials; *$P = 0.012$ for hard trials; $P = 0.19$ for easy versus hard trials, two-sided bootstrap or permutation tests. **g** Difference in miss rate, similar to **d**. ***$P = 8\times10^{-4}$ for easy trials; ***$P = 0.001$ for hard trials; $P = 0.28$ for easy versus hard trials, two-sided bootstrap or permutation tests. **h** Session-by-session correlation between changes in miss rate and error rate for easy (left) and hard (right) trials. Left, $P = 0.02$; right, $P = 0.69$, student's t-test. **i-l** Similar to **a-d**, for control animals (n = 16 sessions, 3 mice) with AAV-Syn-mCherry viral infection. IP injection of CNO itself did not result in increased error rates in control animals. Bootstrapped P values are reported.

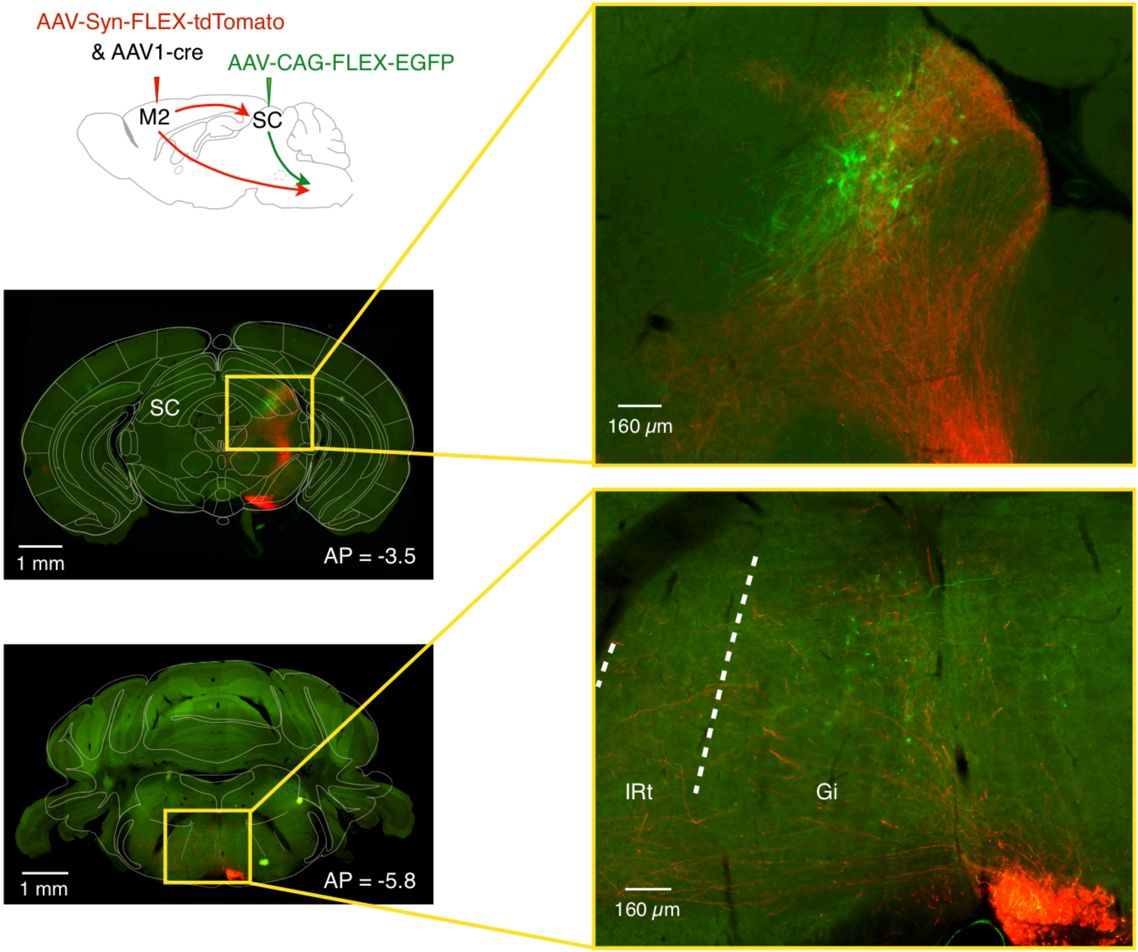

Supplementary Fig. 2 | Descending projections from M2 and lateral SC. To examine the descending projections from M2 and SC to the brainstem, we used anterograde transsynaptic virus to simultaneously label M2 neurons (red) and SC neurons (green) downstream of M2. Red and green axons in the brainstem represent direct projections from M2 and projections from SC neurons downstream of M2, respectively. M2 projections and SC projections overlap in the gigantocellular reticular nucleus (Gi). These data suggest parallel descending pathways for generating licking responses. IRt, intermediate reticular formation.

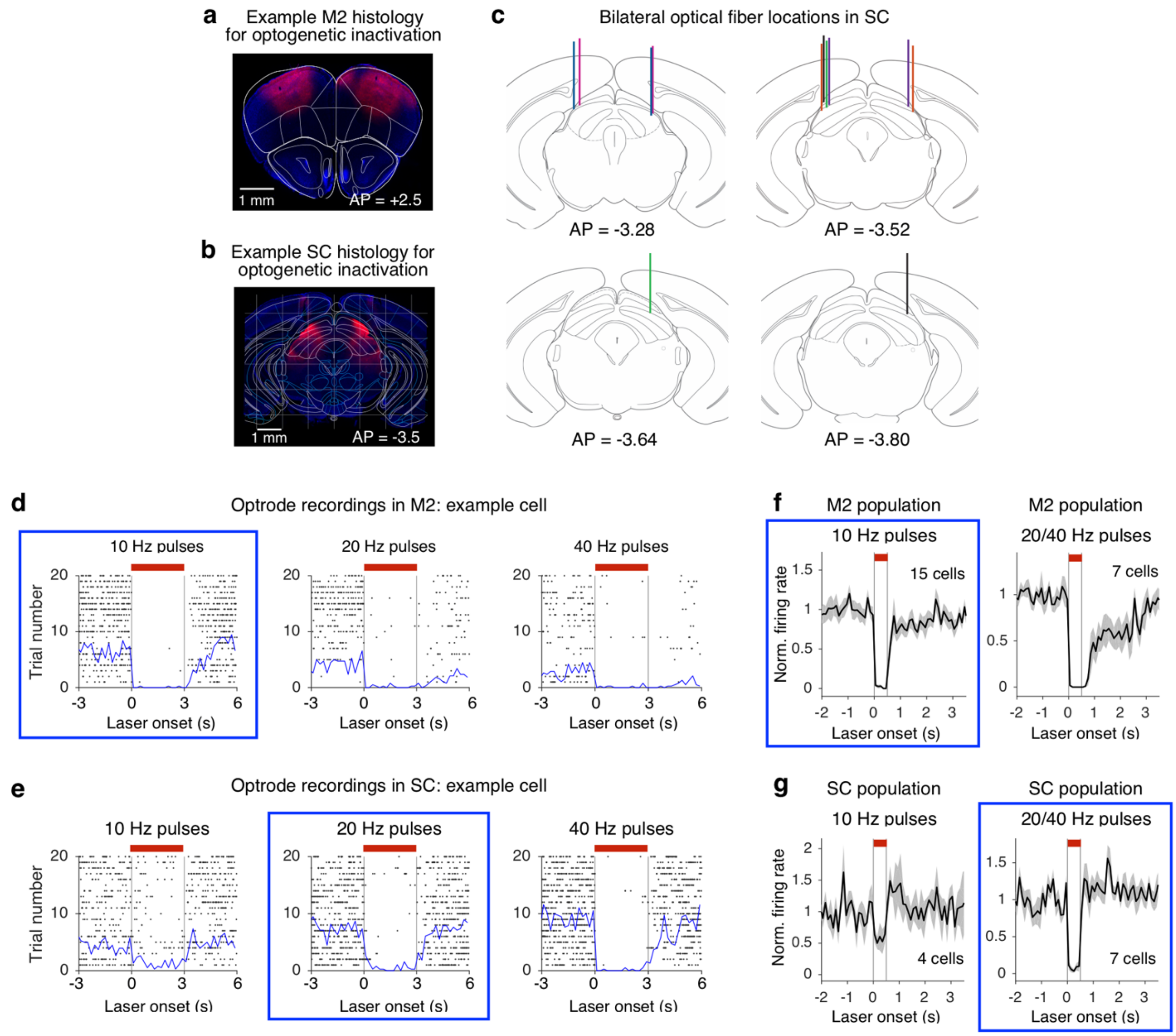

Supplementary Fig. 3 | Optogenetic inactivation in M2 and SC. a-b Example histology of bilateral M2 (**a**) and SC (**b**) infection for optogenetic inactivation. **c** Optical fiber tracks for all mice (n = 6) used for SC optogenetic inactivation experiments. Bilateral fiber tracks from the same animal are labeled with the same color. We positioned the optical fibers to be 200-300 μm above the center of lateral SC virus infection site, -3.5 mm from bregma on the anterior-posterior (AP) axis. The actual fiber tracks were found in coronal sections ranging from -3.80 to -3.28 mm from bregma on the AP axis. **d** Spike activities of an example M2 neuron under 3 types of optogenetic inactivation conditions. Spikes are aligned to laser onset over 20 repeated trials per condition. The red bar marks the laser illumination period (3 s), and the blue line plots the average firing rate as a function of time. In our study, the optimal laser pulse frequency for M2 inactivation is 10 Hz (left panel); higher pulse frequencies resulted in residual inhibition (middle and right panels). **e** Spike activities of an example SC neuron under 3 types of optogenetic inactivation conditions, similar to (**d**). The optimal laser pulse frequency for SC inactivation is 20 Hz (middle panel); lower pulse frequencies resulted in incomplete inhibition (left panel). **f**, **g** similar to **d**,**e**, population data.

a Model 1: P(lick contra) ~ clicks + region + optogenetic epoch + random effects

Model 2: Model 1 + region: optogenetic epoch: difficulty

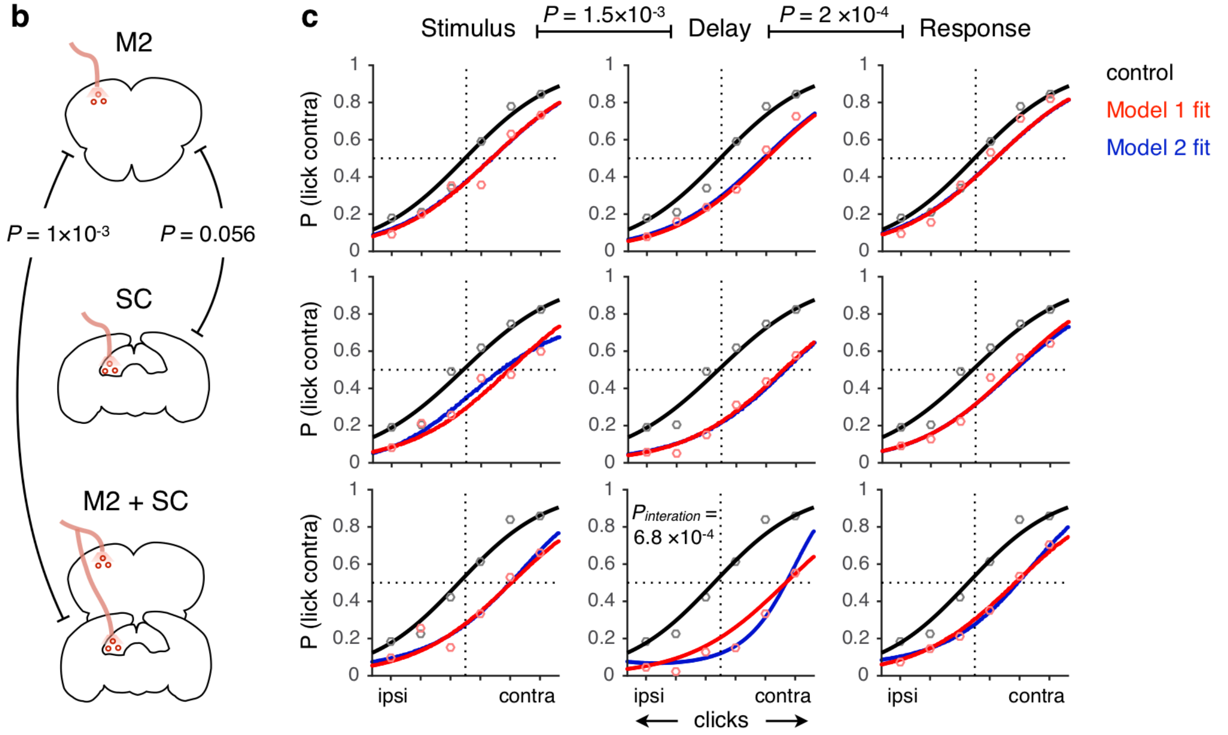

Supplementary Fig. 4 | Simultaneous optogenetic inactivation of M2 and SC. a Pseudo-formula for two GLMMs (logistic functions) used to fit inactivation data. Model 2 includes an extra three-way interaction term compared to Model 1. **b** Schematic for optogenetic inactivation of unilateral M2, SC, or M2 and SC on the same hemisphere. **c** Data (circles, 16391 trials from 36 sessions) and model fit (lines) for optogenetic experiments across different regions and epochs of inactivation. GLMM (model 1, red curves) followed by single-step multiple comparisons (corrected *P* values) reveals a preferential involvement of the cortico-collicular circuit during the delay; and simultaneous inactivation of M2 and SC resulted in a larger impairment than cortical inactivation alone. *P* values report corrected single-step multiple comparison Tukey's test of GLMM results. Model 2 (blue curves) is fit to test which experiment affects difficult trials more compared to easy trials. This interaction term is significant only for simultaneous M2 and SC inactivation during the delay period, and not significant for any other condition (*P*'s > 0.05). *P* values for the interaction term report two-sided z-test results on the coefficients estimated using GLMM, lme4, R.

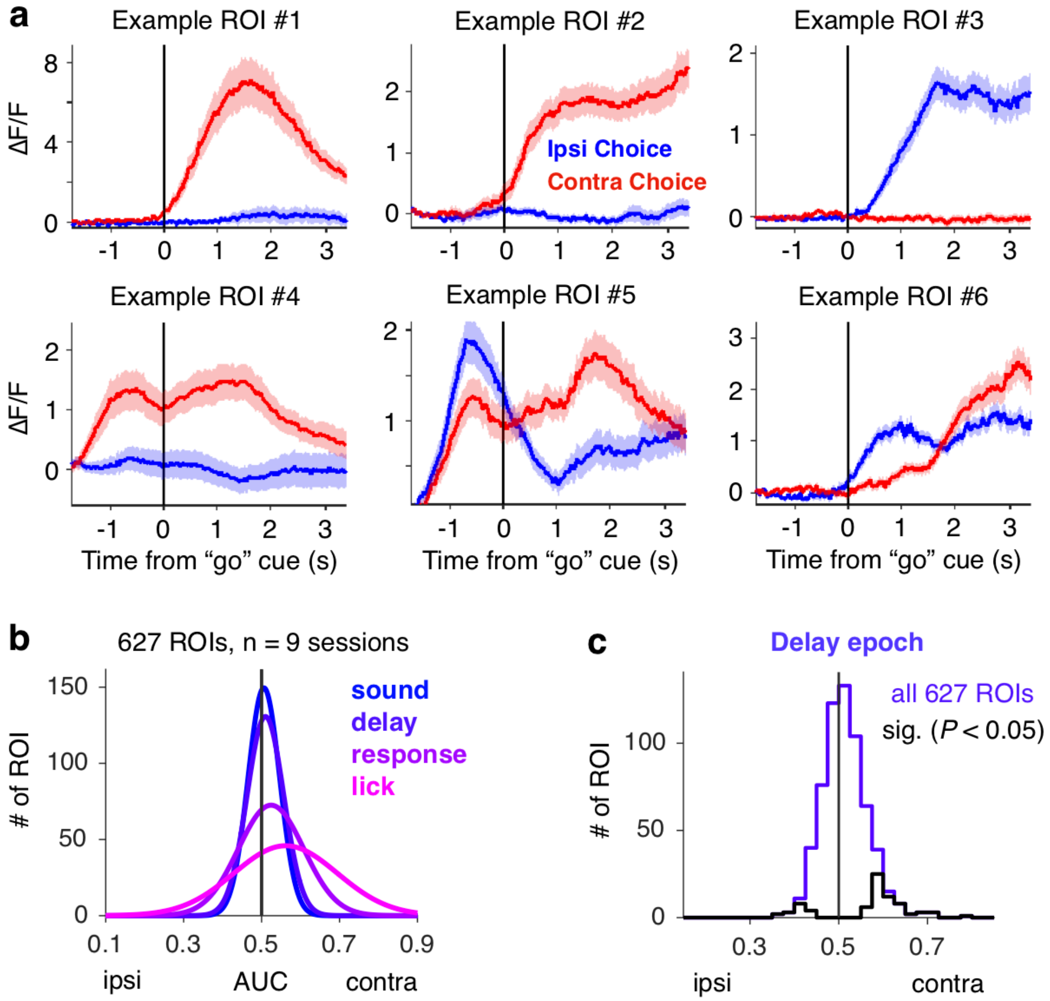

Supplementary Fig. 5 | Single neuron properties of SC-projecting M2 neurons. a Six example SC-projecting M2 neurons. Mean ± s.e.m. ΔF/F traces for contra (red) and ipsi (blue) choice trials, aligned to the “go” cue. **b** Gaussian fits to distributions of choice AUC values of all the ROIs (4 animals) during the sound, delay, response, and lick epochs, similar to Fig. 3**g**. $P = 4\times10^{-4}$, sound versus delay epochs; $P = 4\times10^{-4}$, delay versus response epochs; $P = 4\times10^{-4}$, response versus lick epochs, two-sided permutation tests. **c** Histograms of choice AUC values of all the ROIs (4 animals) during the delay epoch. Black lines show individually significant neurons, two-sided bootstrapped $P's < 0.05$.

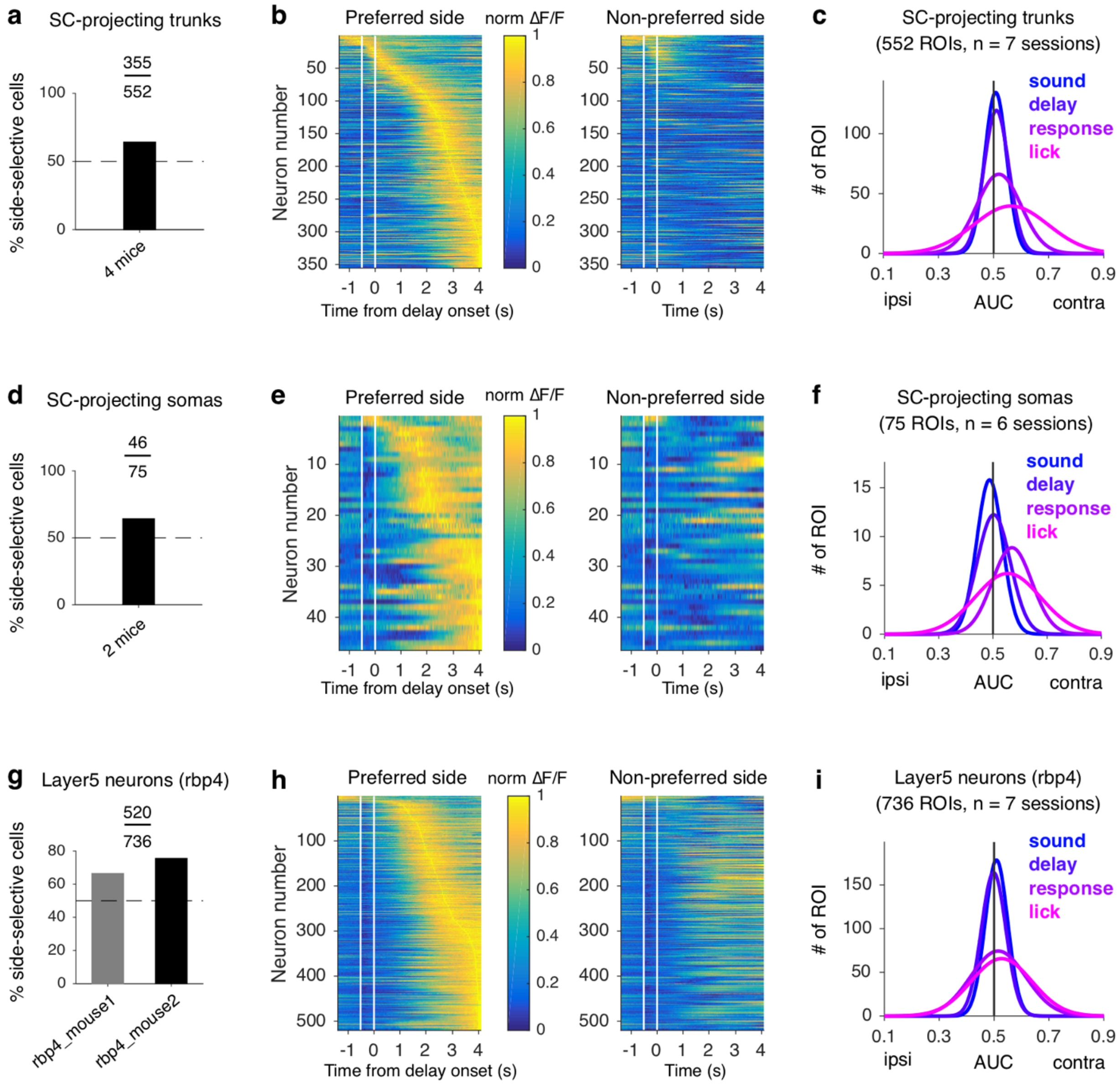

Supplementary Fig. 6 I Response properties in SC-projecting M2 trunks, somas, and generally labeled M2 layer 5 neurons. a-c Choice-selectivity in SC-projecting M2 trunks, similar to Fig. 3b,c,g. **d-f** Choice-selectivity in SC-projecting M2 somas, similar to Fig. 3b,c,g. **g-i**, Choice-selectivity in generally labeled layer 5 pyramidal neurons in M2, with no projection specificity, similar to Fig. 3b,c,g.

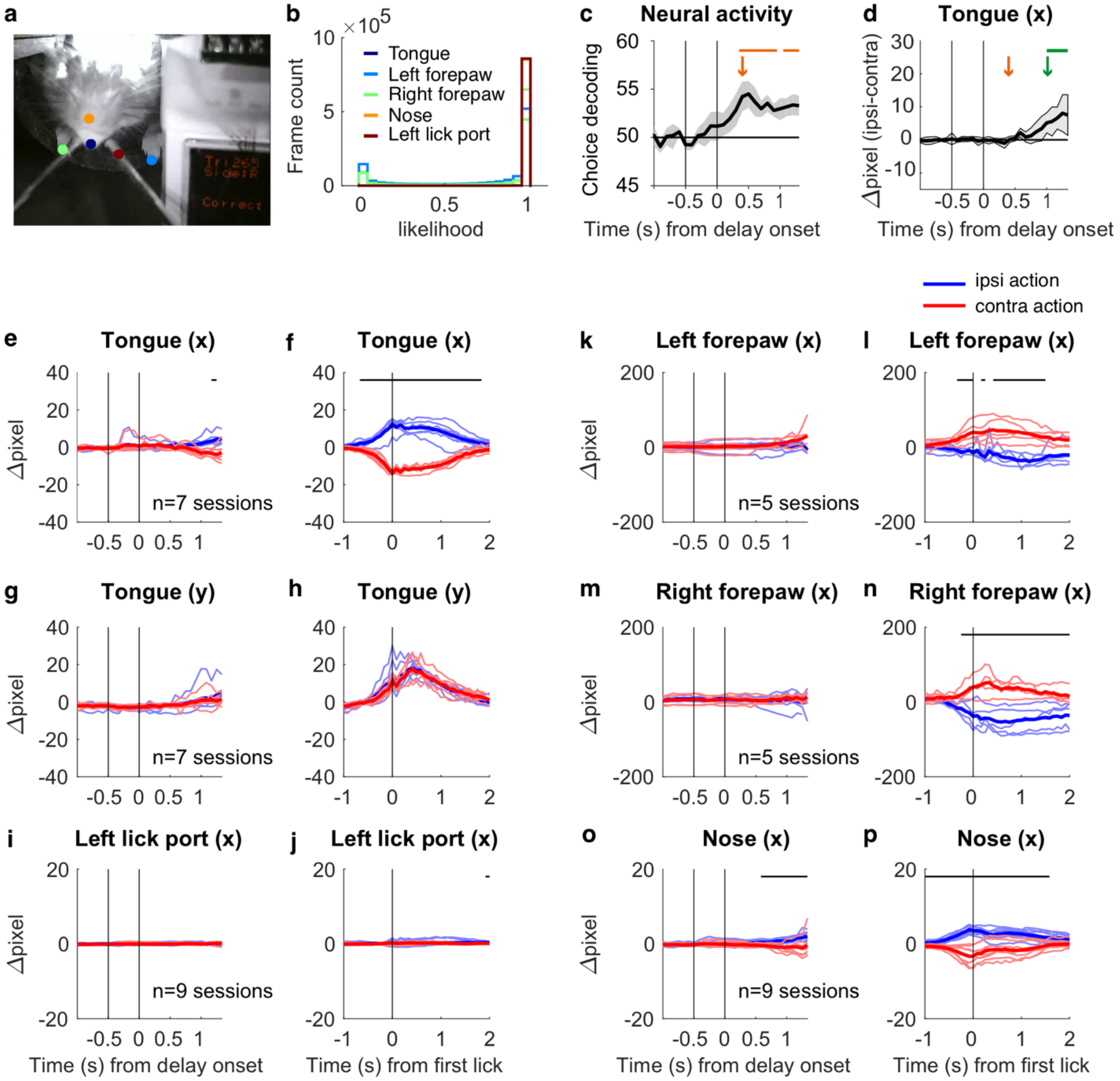

Supplementary Fig. 7 | Video analyses during two-photon calcium imaging. a Image of example video data during two-photon calcium imaging with 5 movement variables used in the model, indicated as colored dots. **b** Distribution of likelihood for trained DeepLabCut model results for the 5 movement variables across frames. **c** Classification accuracy of decoders trained on simultaneously recorded neurons to differentiate between left versus right choices across imaging frames, aligned to delay onset. Mean ± s.d. across 7 imaging sessions. Timing of significant decoding above chance ($P < 0.01$, bootstrap, two-sided) is indicated by the orange arrow and bar above. **d** Choice selectivity based on tracked tongue movements (Δ pixel from baseline position) across video frames. Mean ± s.d. across imaging sessions. Timing of significance ($P < 0.01$, bootstrap, two-sided) is indicated by the green arrow and bar above. Orange arrow is plotted to show the earliest timing of significant choice selectivity from neural

data, for comparison. **e**, **f** Tracked horizontal tongue movements for ipsi- (blue) and contra-action (red) trials during the stimulus and delay period (**e**), and response period (**f**). Timing of significant difference ($P < 0.01$, bootstrap, two-sided) between ipsi- and contra-lateral tongue movements is indicated by the bar above. Thick line, mean across sessions; thin lines, individual sessions. **g-p** similar to **e**,**f**, for vertical tongue movement (**g**,**h**); left lick port (**i**,**j**); horizontal left forepaw movement (**k**,**l**); horizontal right forepaw movement (**m**,**n**); horizontal nose movement (**o**,**p**).

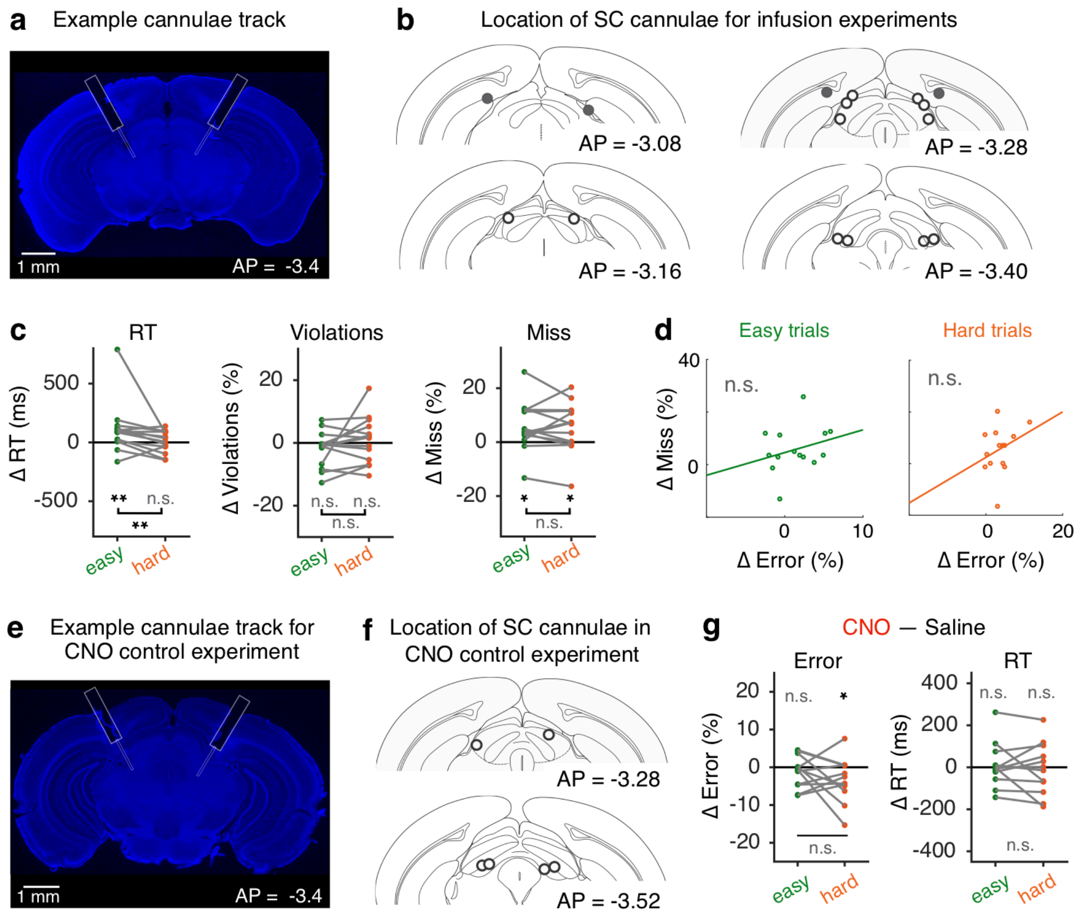

Supplementary Fig. 8 | Pathway-specific inactivation of M2 terminals in SC. a Example cannulae track for SC infusion experiment. **b** Histology of bilateral SC cannulae placement for all mice implanted for M2 terminal inhibition experiments (n = 8 mice). Open and closed circles mark the locations of the injector tips that were within the SC or outside the SC, respectively. Six out of 8 mice that underwent surgeries had cannulae placed correctly within the SC, and were included for analyses in the M2 terminal inhibition experiment. **c** Difference in RT (left), violation rate (middle), and miss rate (right) between M2 terminal inhibition sessions (n = 14) and saline control sessions (n = 14) for easy (green) and hard (orange) trials. Gray lines connect data from the same session. Left, $^{**}P = 0.0076$ for easy trials; $P = 0.74$ for hard trials; $^{**}P = 0.0032$ for easy versus hard trials. Middle, $P = 0.17$ for easy trials; $P = 0.63$ for hard trials; $P = 0.06$ for easy versus hard trials. Right, $^{*}P = 0.013$ for easy trials; $^{*}P = 0.027$ for hard trials; $P = 0.86$ for easy versus hard trials. Two-sided bootstrap or permutation tests. **d** Session-by-session correlation between changes in miss rate and error rate for easy (left) and hard (right) trials after chemogenetic inactivation of M2 terminals in SC. Student's t-test. **e-g** Similar to **a-c**, for control animals (n = 12 sessions, 3 mice) with AAV-Syn-mCherry viral infection in M2 neurons. **g** Left panel, $^{*}P = 0.028$, two-sided bootstrap.

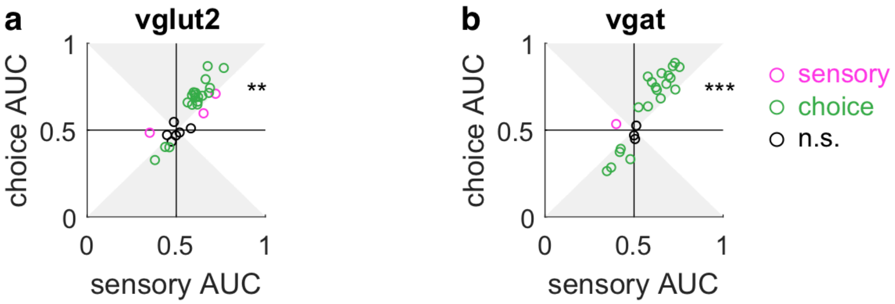

Supplementary Fig. 9 I Side-selective activity in SC neurons encode the upcoming choice. a Sensory versus choice AUC values for all sessions in SC excitatory populations (Vglut2) during the late epoch. AUC values below or above 0.5 indicate preference for ipsi- or contra-actions, respectively. Green circles, sessions with significant AUC values, higher for choice encoding. Magenta, higher for sensory encoding. Black, sessions with non-significant AUC values. Shaded areas indicate the range where choice AUC values are larger than sensory AUC values. $^{**}P = 0.0016$, Wilcoxon signed rank test across sessions, two-sided. **b** similar to **a**, for all sessions in SC inhibitory populations (Vgat). $^{***}P = 5.6\times10^{-5}$, Wilcoxon signed rank test across sessions, two-sided.

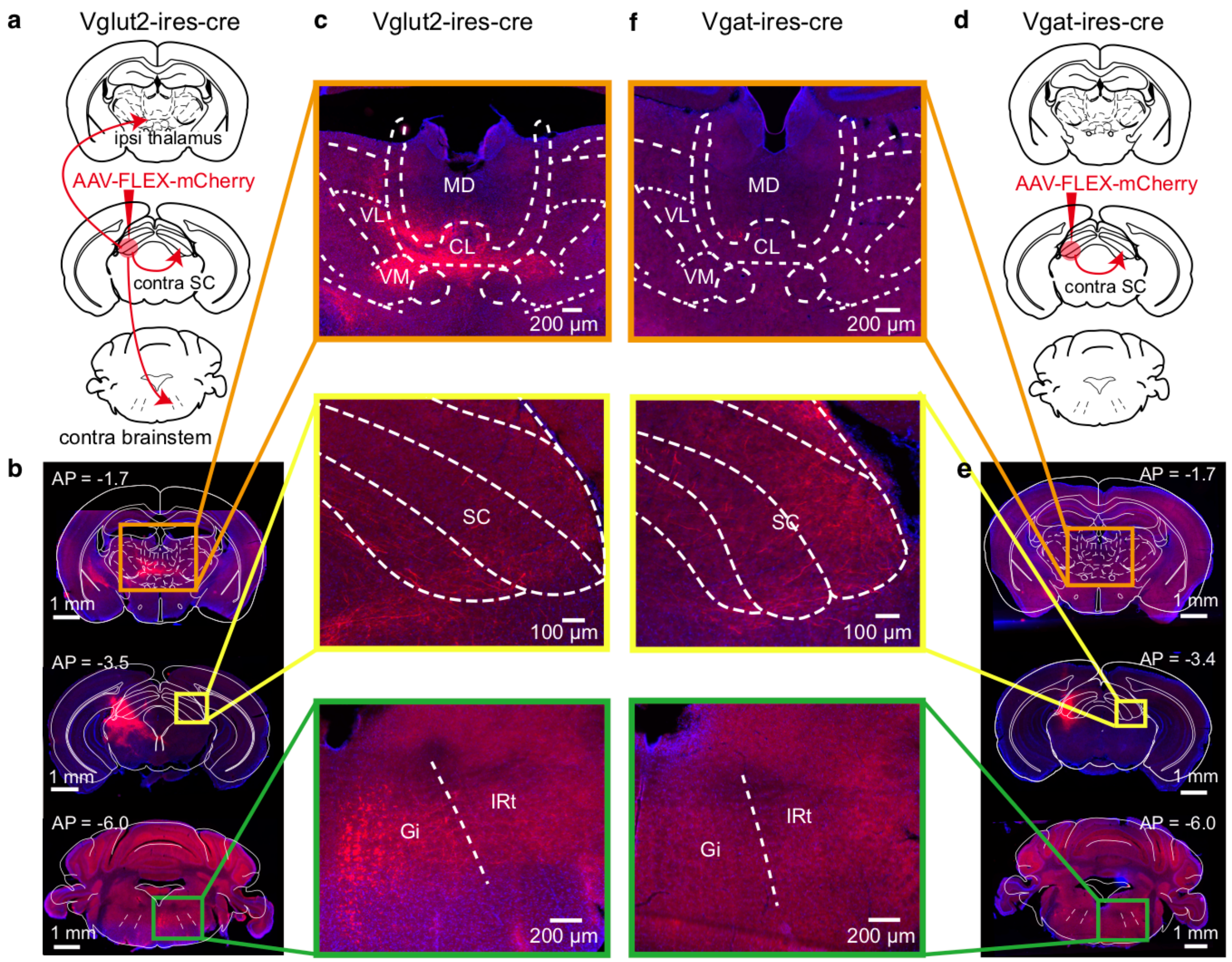

Supplementary Fig. 10 l Projection patterns of SC excitatory and inhibitory neurons. a-c Anterograde tracing from SC excitatory neurons reveals projection terminals in ipsilateral thalamic nuclei, contralateral SC, and contralateral brainstem nuclei (Gi and IRt). **d-f** similar to **a-c**, for SC inhibitory neurons, which also project to the contralateral SC, but avoid the thalamus and brainstem.
