# Supplementary material for: A cortico-collicular pathway for motor planning in a memory-dependent perceptual decision task
Source: Nat Commun. 2021 May 11;12:2727. doi: 10.1038/s41467-021-22547-9 (PMC8113349; doi:10.1038/s41467-021-22547-9)
Supplement: Supplementary file 3 — Reporting Summary [file 41467_2021_22547_MOESM3_ESM.pdf]

nature research

Corresponding author(s): Chunyu A. Duan and Ning-long Xu

Last updated by author(s): Mar 1, 2021

Reporting Summary

Nature Research wishes to improve the reproducibility of the work that we publish. This form provides structure for consistency and transparency in reporting. For further information on Nature Research policies, see our Editorial Policies and the Editorial Policy Checklist.

Statistics

For all statistical analyses, confirm that the following items are present in the figure legend, table legend, main text, or Methods section.

n/a	Confirmed	
☐	☒	The exact sample size (*n*) for each experimental group/condition, given as a discrete number and unit of measurement
☐	☒	A statement on whether measurements were taken from distinct samples or whether the same sample was measured repeatedly
☐	☒	The statistical test(s) used AND whether they are one- or two-sided<br>*Only common tests should be described solely by name; describe more complex techniques in the Methods section.*
☒	☐	A description of all covariates tested
☐	☒	A description of any assumptions or corrections, such as tests of normality and adjustment for multiple comparisons
☐	☒	A full description of the statistical parameters including central tendency (e.g. means) or other basic estimates (e.g. regression coefficient) AND variation (e.g. standard deviation) or associated estimates of uncertainty (e.g. confidence intervals)
☐	☒	For null hypothesis testing, the test statistic (e.g. F, t, r) with confidence intervals, effect sizes, degrees of freedom and P value noted<br>*Give P values as exact values whenever suitable.*
☒	☐	For Bayesian analysis, information on the choice of priors and Markov chain Monte Carlo settings
☒	☐	For hierarchical and complex designs, identification of the appropriate level for tests and full reporting of outcomes
☒	☐	Estimates of effect sizes (e.g. Cohen's d, Pearson's r), indicating how they were calculated

Our web collection on statistics for biologists contains articles on many of the points above.

Software and code

Policy information about availability of computer code

Data collection	Custom code based on Python (v2.7); Arduino IDE (1.5.6r2); ScanImage (4.2); Micro-Manager (2.0 beta).
Data analysis	Custom code written in MATLAB (R2015b); custom code written in RStudio (1.0.143); ImageJ (1.53c).

For manuscripts utilizing custom algorithms or software that are central to the research but not yet described in published literature, software must be made available to editors and reviewers. We strongly encourage code deposition in a community repository (e.g. GitHub). See the Nature Research guidelines for submitting code & software for further information.

Data

Policy information about availability of data

All manuscripts must include a data availability statement. This statement should provide the following information, where applicable:

- Accession codes, unique identifiers, or web links for publicly available datasets
- A list of figures that have associated raw data
- A description of any restrictions on data availability

All data to understand and assess the conclusions of this study are available in the main text or supplementary materials. All the original behavioral, optogenetic, chemogenetic, electrophysiological, imaging, and tracing data are archived in the Institute of Neuroscience, Chinese Academy of Sciences, and can be obtained upon reasonable request via email to the corresponding authors.

Field-specific reporting

Please select the one below that is the best fit for your research. If you are not sure, read the appropriate sections before making your selection.

☒ Life sciences ☐ Behavioural & social sciences ☐ Ecological, evolutionary & environmental sciences

For a reference copy of the document with all sections, see nature.com/documents/nr-reporting-summary-flat.pdf

Life sciences study design

All studies must disclose on these points even when the disclosure is negative.

Sample size	Unless indicated in the Methods, no statistical methods were used to pre-determine sample sizes. Sample sizes were instead determined by the limits of data collection within a reasonable time, and are similar to those reported in previous publications (Refs 7-11). All groups had similar sample sizes. In this study, 9 mice were used for optogenetic inactivation and control experiments; 11 mice were used for chemogenetic inactivation and control experiments; 5 mice were used for two-photon calcium imaging experiments; 6 mice were used for whole-cell recordings in brain slices; 11 mice were used for fiber photometry recording experiments; and 15 mice were used for tracing studies and immunohistochemistry.
Data exclusions	Data from 2 mice were excluded from analyses for projection-specific chemogenetic inactivation based on histology (Methods).
Replication	Results in chemogenetic (8 mice) and optogenetic inactivation experiments (6 mice) were consistent across individual animals tested. These two independent methods reached similar conclusions regarding the hypotheses tested. Two-photon calcium imaging experiments were carried out from 5 mice, over multiple imaging planes. Fiber-photometry experiments were carried out from 11 mice. For whole-cell recordings in brain slices, 24 excitatory cells from 2 mice and 44 inhibitory cells from 4 mice were recorded. All anatomical tracing experiments were conducted in multiple animals per condition.
Randomization	Animals used in control and testing groups were randomly chosen from the common pool of purchased animals or in-house bred litters.
Blinding	All behavioral and neural responses in the in vivo and in vitro experiments were objectively measured by automated hardware and software system that do not require human intervention, and therefore were not blinded to investigators.

Reporting for specific materials, systems and methods

We require information from authors about some types of materials, experimental systems and methods used in many studies. Here, indicate whether each material, system or method listed is relevant to your study. If you are not sure if a list item applies to your research, read the appropriate section before selecting a response.

Materials & experimental systems

n/a	Involved in the study	
☐	☒	Antibodies
☒	☐	Eukaryotic cell lines
☒	☐	Palaeontology and archaeology
☐	☒	Animals and other organisms
☒	☐	Human research participants
☒	☐	Clinical data
☒	☐	Dual use research of concern

Methods

n/a	Involved in the study	
☒	☐	ChIP-seq
☒	☐	Flow cytometry
☒	☐	MRI-based neuroimaging

Antibodies

Antibodies used	Primary antibody: anti-GABA (rabbit, dilution 1:1000, A2052; Sigma); secondary antibody: donkey anti-rabbit (Alexa Fluor 594, dilution 1:1000, A-21207; ThermoFisher Scientific).
Validation	The primary antibody we used in this study has been validated in mouse brain slices in the following peer-reviewed papers:<br><br>1. Glickstein, S. B., Moore, H., Slowinska, B., Racchumi, J., Suh, M., Chuhma, N., & Ross, M. E. (2007). Selective cortical interneuron and GABA deficits in cyclin D2-null mice. Development (Cambridge, England), 134(22), 4083–4093. doi:10.1242/dev.008524<br>2. Tasan, R. O., Bukovac, A., Peterschmitt, Y. N., Sartori, S. B., Landgraf, R., Singewald, N., & Sperk, G. (2011). Altered GABA transmission in a mouse model of increased trait anxiety. Neuroscience, 183, 71–80. doi:10.1016/j.neuroscience.2011.03.051

Animals and other organisms

Policy information about studies involving animals; ARRIVE guidelines recommended for reporting animal research

Laboratory animals	Species: Mouse.<br>Strain: C57BL/6J, Vgat-IRES-Cre, Vglut2-IRES-Cre, Rbp4-Cre, Vglut2-ires-Ai9, Vgat-ires-Ai9.<br>Sex: male.<br>Age: 8 weeks old from the beginning of experiments.<br><br>All mice were housed under standardized conditions, with temperature controlled between 21-23 ˚C and humidity controlled between 40-70%.
Wild animals	The study did not involve wild animals.
Field-collected samples	The study did not involve field-collected samples.
Ethics oversight	All animal use procedures were approved by the Animal Care and Use Committee of the Institute of Neuroscience, Chinese Academy of Sciences.

Note that full information on the approval of the study protocol must also be provided in the manuscript.
